# Data extraction for complex meta-analysis (DECiMAL) guide

**DOI:** 10.1186/s13643-016-0368-4

**Published:** 2016-12-13

**Authors:** Hugo Pedder, Grammati Sarri, Edna Keeney, Vanessa Nunes, Sofia Dias

**Affiliations:** 1 National Guideline Alliance, Royal College of Obstetricians and Gynaecologists, London, UK; 2 Evidera Inc., London, UK; 3 School of Social and Community Medicine, University of Bristol, Bristol, UK

**Keywords:** Meta-analysis, Data, Extraction, Guide, Analysis, Review, Network, Multivariate

## Abstract

As more complex meta-analytical techniques such as network and multivariate meta-analyses become increasingly common, further pressures are placed on reviewers to extract data in a systematic and consistent manner. Failing to do this appropriately wastes time, resources and jeopardises accuracy. This guide (data extraction for complex meta-analysis (DECiMAL)) suggests a number of points to consider when collecting data, primarily aimed at systematic reviewers preparing data for meta-analysis. Network meta-analysis (NMA), multiple outcomes analysis and analysis combining different types of data are considered in a manner that can be useful across a range of data collection programmes. The guide has been shown to be both easy to learn and useful in a small pilot study.

**Electronic supplementary material:**

The online version of this article (doi:10.1186/s13643-016-0368-4) contains supplementary material, which is available to authorized users.

## Background

Data collection is a vital part of a systematic review. It bridges the gap between a review and a meta-analysis. Making this as easy, understandable and accurate as possible hugely speeds up the process of data cleaning and checking for the data analyst/reviewer. Lack of co-ordination between reviewers and analysts can lead to errors which may feed through to produce incorrect results and inferences in systematic reviewing.

As more complex techniques such as network and multivariate meta-analyses become increasingly common in systematic reviews, further demands are placed on reviewers to extract data in a systematic and consistent manner. Learning from the experience on conducting systematic reviews and complex meta-analyses to inform decision-making for the development of UK National Institute for Health and Care Excellence (NICE) guidelines, this guide was developed after discussions with senior reviewers, with the intention of improving the consistency and accuracy of data collection.

Further development and initial testing of the usefulness of this guide was performed in a pilot study involving reviewers from two UK NICE clinical guideline development teams and centres. Reviewers with a wide range of experience in systematic reviewing from across the centres were invited to participate in the study. Fifteen out of 25 reviewers (60% response rate) completed two mock data extractions (one network meta-analysis (NMA) and one multivariate extraction) and then evaluated the guide using a modified version of the 10-item System Usability Scale [1]. Feedback from reviewers was used to further improve the guide.

An initial review of available data extraction guides in systematic reviewing identified a paucity of tools to guide data collection for complex evidence synthesis. Brown et al. report on a framework for developing a coding scheme for data extraction for meta-analysis, but the authors did not cover the more technical issues that can arise during complex meta-analysis, such as multiple arms and correlated outcomes [2]. We also identified several data extraction templates developed by the Cochrane Collaboration which provides guidance on topics to be covered in data extraction and quality assessment at a study level but does not suggest methods for organising multiple studies [3].

In order to cover this gap in the literature, we have developed a guide (data extraction for complex meta-analysis (DECiMAL)) to assist reviewers extracting data from systematic reviews in a consistent way for use in meta-analyses. The guide was not designed with the aim to be exhaustive but to address most of the problems faced when collecting various types of data, such as time-to-event, binary or continuous, for complex analyses such as NMA and multivariate meta-analyses. Since it is much easier to identify and correct data collection issues before all data are collected, this guide aims to raise early awareness of these issues so that they can be discussed and addressed from the outset of the process.

This guide is intended to assist reviewers only with the data extraction aspects of meta-analysis. It does not provide instructions on statistical techniques of meta-analysis in systematic reviews, such as handling of missing data or converting summary statistics, as reviewing them is not the aim of this paper. It also is intended to assist only with data extraction for aggregate data meta-analyses, as methods will differ for individual patient data meta-analyses.

Many different database programmes are available for managing data. Microsoft Excel or Microsoft Access are often used for smaller datasets, whilst more specific statistical software, such as STATA or R, may be used for larger projects which require more complex data manipulation. Some software will have inbuilt functions that restrict input to certain types of data, such as string or numerical, depending on how each variable has been pre-specified. For instance, programmes such as Review Manager already have built-in functions to address many of the issues discussed in this guide, though as a result, the procedures for analysis are more limited.

The points suggested here will be relevant for almost any software that is used for data collection, provided they can be visualised in the format of rows of observations (studies in this case) and columns of variables.

The guide is structured as follows:

- *The “* *Background* *” section contains information on data extraction for different types of analysis*
  - ◦ *Suggestions 1–4* apply mainly to data collection for network meta-analysis
  - *◦ Suggestions 5–6* describe issues with data collection involving multiple outcomes which may inform a multivariate meta-analysis
- *The “* *Discussion* *” section contains information on data extraction for different types of data*
  - *◦ Suggestions 7–14* describe ways of collecting data of different types, such as time-to-event data or relative effect data
- *The “* *Conclusions* *” section contains general information on data extraction*
  - *◦ Suggestions 15–27* make some general points reviewers should be aware of, regardless of the type of data or meta-analysis their data collection will inform.
- *Additional file* *1* *is an Excel workbook containing five worksheets:*
  - *◦* One study per row (arm): example data extraction for a meta-analysis of arm-based (absolute) data in the one study per row format
  - *◦* One study per row (relative): example data extraction for a meta-analysis of relative data in the one study per row format
  - *◦* Rate data: example data extraction for a meta-analysis of rate data in the one study per row format
  - *◦* Diagnostic test accuracy: example data extraction for a diagnostic test accuracy meta-analysis
  - *◦* Codebook: example of a glossary worksheet to demonstrate the coding of different variables in a data extraction

### DECiMAL guide

#### Data extraction for different types of analysis

##### Network meta-analysis

1. When collecting data for a network meta-analysis (NMA), always note in a separate numerical column how many arms the trial had.
  1.1. Also (in another column) note the arm number that the observation/row in the database refers to and keep these consistent when collecting data with multiple outcomes or at multiple time points (e.g. keep placebo in arm 1 for all outcomes).
2. Decide on a sensible treatment numbering and classification in advance. This will help with correctly numbering the arms when extracting data. By ensuring that the highest numbered treatment is always compared to the lowest, the effect estimates will be consistent (Additional file 1 — Codebook).
3. Different combinations or doses of interventions can be added as separate treatments, with separate numbers/classifications to distinguish between them, depending on how the protocol specifies these should be analysed.
4. A one study per row format can be useful to prevent duplication of study ID, treatments, numbers randomised and other characteristics (e.g. risk of bias), provided the data are not too complex.
  4.1.  Multiple outcomes and time points can be collected onto the same row in new columns (though this can become cumbersome with many time points and outcomes).
5. It can be easier to collect arm-based (absolute) data on one worksheet and relative data on a different worksheet, since they will require different columns and different analysis approaches (Additional file 1—One study per row (arm) and One study per row (relative)).
  5.1.  For relative effects, extra columns will be needed to clarify which treatment is being compared to which. Care should be taken to identify which treatment is the “comparator” and which is the “experimental” (*see Suggestion 19*).
  5.2.  When extracting relative effects for ratio outcomes, these should be extracted on the natural-logarithm scale (e.g. log-hazard ratios) with their standard errors.

##### Multiple outcomes and multivariate meta-analysis

5. These can either be collected with a separate row for each outcome, or (preferably) in the one study per row format, with an additional set of columns for each additional outcome (Additional file 1—One study per row (arm) and one study per row (relative)).
6. Multiple time points can be extracted similarly to multiple outcomes, with each time point from the same study extracted as either a separate row or in the one study per row format.
7. Joint distributions may be reported in some studies—this is where the number of patients with each outcome is reported for each level of another outcome.
  7.1.  For instance, “gestational age” and “mode of birth” are reported as outcomes. Their joint distribution can be obtained if gestational age is reported separately for each mode of birth (e.g. vaginal: mean = 39.5 weeks, SD = 5 weeks; caesarean: mean = 40.7 weeks, SD = 4.7 weeks).
  7.2.  If data for joint distributions are reported, then a simple note that this is the case should be written consistently in a notes column, as this information can be used for multivariate meta-analysis or for health economic modelling (Additional file 1 — Rate data). The full data can then be extracted more easily at a later date when and if it is needed.
  7.3.  Diagnostic accuracy studies should be analysed using a multivariate approach to account for the correlation between sensitivity and specificity. The numbers of true positives, false positives, true negatives and false negatives should be extracted into separate columns for each study (Additional file 1 — Diagnostic test accuracy). Care must be taken to ensure which is the reference and which the index test.
    7.3.1.  Where 2 × 2 tables of true positives, false positives, true negative and false negatives are not reported in the original studies, these can be calculated from sensitivity and specificity providing the overall number of participants and the total number of participants that tested positive on either the index or the reference test are available.

#### Data extraction for different types of data

##### Time-to-event data (e.g. recurrence of cancer)

7. Hazard ratios and their measure of uncertainty should always be collected where available.
8. It should be noted if Kaplan-Meier plots or life tables are reported (add a new column to indicate if a study reports this), as methods are available to reconstruct individual participant data from these.

##### Rate data (e.g. frequency of migraine episodes)

9. When rates are reported, the total number of person-years at risk should also be collected (Additional file 1—Rate data).
10.  If this is not available, then the average length of follow-up and the total number of patients at the end of the study should be collected instead, as these can be used to approximate the total person-years (by making some extra assumptions).
11.  Sometimes, rate data are reported either as the number of first events or the total number of events, in a given time period. It is important to distinguish between these as they may need to be modelled separately. This can be done by having separate columns to collect each type of data (usually the most appropriate option), or by including a column which states which data type it is.

##### Binary and categorical variables (Additional file 1 – One study per row (arm))

12.  If you are dealing with binary responses, it is normally easier to use numbers than letters or text (Additional file 1—One study per row (arm)).
  12.1.  For yes/no responses, use 1 for yes and 0 for no.
  12.2.  For other responses that do not have a clear response direction, use 1 and 2 and state (in the variable name in the first row or in a glossary worksheet) which number corresponds to which category—e.g. age_strata (1 ≤ 55 years, 2 ≥ 55 years) (Additional file 1—One study per row (arm): *stroketype* variable).
    12.2.1.   We leave the choice of whether to use a glossary worksheet/codebook (Additional file 1 — Codebook) or to refer to the code within the variable name itself (Additional file 1 — Diagnostic test accuracy) up to the individual reviewer. Longer but more detailed variable names will help with data extraction but can create difficulties during data analysis.
    12.2.2.   A further alternative is to add an additional row below the variable name to hold the short code for that variable. This row can be hidden during data extraction or analysis if desired.
  12.3.  Both numbers of patients randomised and those that complete the trial should be extracted, along with the numbers that discontinued treatment grouped by the reason for discontinuation (e.g. due to adverse events) (Additional file 1 — One outcome per row (arm): *disc* and *discAE* variables). These numbers can be useful for dealing with missing data, for example using sensitivity analyses.

##### Continuous and ordinal variables

13.  When working with mean differences, both final values and change from baseline in primary studies can be combined if baselines within a trial are equal (as they should be in a randomised trial). Treatment differences should be the same irrespective of which measure is reported. However, change from baseline is preferable to final values if both are reported in a study. Baseline values should also be extracted if available (Additional file 1—One study per row (relative)).
  13.1.  For example, one study may report mean change from baseline for systolic blood pressure in both the active and reference group (active = −5 mmHg from baseline, reference = −1 mmHg from baseline). This can be meta-analysed directly with a study that only reports mean final systolic blood pressure values in each group (active = 118 mmHg, reference = 124 mmHg), provided that the baselines are equal, as the treatment difference will be the same (mean difference for change from baseline = −5 − −1 = −4 mmHg, mean difference for final values = 118 − 124 = −6 mmHg).
14.  Keep units consistent
  14.1.  Ideally choose a consistent unit to report all instances of a particular variable (e.g. months, mg/day).
  14.2.  If many different units are reported and it feels like a lot of effort to constantly work out a consistent unit, do not waste too much time doing this — it is easy to do afterwards when analysing the data. Simply make a new column alongside the variable and state the units for each number (Additional file 1 — Rate data: *dose* and *dose_unit* variables)
  14.3.  Similarly, this type of coding can be used if there are multiple scales for one outcome (e.g. pain, anxiety) (Additional file 1 — One study per row (relative): *scale* variable)

#### General points

15.  Ask questions
  15.1.  If unsure about how a particular variable should be entered into a spreadsheet, ask the data analyst the format they would like it in.
  15.2.  It is much easier to be able to identify and correct an issue before all the data are collected than to try to change it afterwards.
16.  Be consistent
  16.1.  The most important thing when collecting data is to be consistent about how outcomes are entered into a spreadsheet.
  16.2.  Keep data entries in the same case (lower case is easiest for everyone…do not worry about it looking less pretty).
  16.3.  Preferably choose text items from a pre-specified list that you can programme into the software you are using.
17. Use short abbreviations for naming variables, and record these in a glossary page
  17.1.  Use easily recognisable abbreviations where possible (e.g. “L95” for lower 95% CI or “narm” for the number of treatment arms in a study).
  17.2.  A separate worksheet in the file can then be used as a glossary page for the column/variable names, indicating what each abbreviation means and what each code in the column/variable represents (e.g. for treatment classification numbers; 1 = placebo, 2 = nifedipine, 3 = ritodrine) (Additional file 1 — Codebook).
18.  Record study and participant characteristics that could help explain between-study heterogeneity
  18.1.  These can be added in additional columns where necessary and should ideally be specified a priori in a review protocol.
19.  Do not leave blank cells
  19.1.  If a value is not reported, use “NR”, rather than leaving a cell blank; otherwise, it is not clear if the value is not reported in the study or if you forgot to write it down.
  19.2.  If a value is not applicable for a particular study, write “NA”.
  19.3.  If possible, set up your data collection form so that no blank cells are allowed.
20.  Do not include a space before or after a cell value
  20.1.  Ensure that each time a value is entered into a cell, there are no blank spaces before or after the value. This is important as any studies that contain values with blank spaces may be excluded when importing data to other software.
21.  Consider the direction of effect
  21.1.  When entering effect measures, consider which treatment is the numerator (active treatment) and which is the denominator (reference treatment) in ratio measures, or which treatment is subtracted from which in difference measures.
    21.1.1.   In placebo-controlled trials, this should be obvious, but if one drug is compared to another, the direction may be different to what you expect.
    21.1.2.   When extracting relative effects (e.g. hazard ratios, odds ratios, mean differences), it is easier to always use the treatment with the highest treatment classification number (see 17.2) as the active treatment (Additional file 1 — Codebook).
  21.2.  Take care when extracting “reduction” or “increase” outcomes as sometimes a reduction of e.g. 3.2 units may be reported as “–3.2” or as “reduction of 3.2”. The correct sign needs to be extracted and kept consistent across primary studies. If in doubt double-check tables and text to ensure the direction is correctly extracted.
22.  Avoid mixing words (“*string*”) and numbers (“*numerical*”) in the same cell unless absolutely necessary
  22.1.  This includes putting commas in numbers (e.g. write 10000 rather than 10,000)
  22.2.  If you want to annotate a particular numerical value or study you have entered, add the annotation in a new column alongside the existing variable (Additional file 1 — Rate data: *notes* variable)
23.  Avoid colour coding
  23.1.  It is usually not possible to import data into statistical programs based on colour coding. Therefore, it is usually more useful to add an additional notes column to identify a particular row of data.
24.  Consistency when working with others
  24.1.  If working with another reviewer to extract data into the same spreadsheet, ensure that you know exactly how they have coded their variables, so as to keep responses consistent. This can be achieved by working using the same glossary/code book for reference, which should ideally be prepared before the data extraction, based on the review protocol.
  24.2.  If unsure, ask the other reviewer how they may have dealt with a particular study/outcome.
25.  Keep text cells to a minimum
  25.1.  Avoid text where numbers or a classification code could be used instead (*see Suggestion 11 — Binary variables*).
  25.2.  If text cells must be used, then it is better to pre-define all possible values and select them from a list rather than free-typing them each time (which could lead to errors).
26.  Uncertainty and variability
  26.1.  Report SEs, SDs, and 95% confidence limits in separate columns.
  26.2.  If none of these are available, report a *p* value if its exact value is given (*p* = 0.024 rather than *p* < 0.05) and add a variable to note which statistical test the *p* value is based on (e.g. *t* test, log-rank test). These can be used to calculate variability in some circumstances.
27.  Data checking for accuracy
  27.1.  A proportion of the data extraction (ideally 100%) should be repeated by a second reviewer, or at least a random check of the extraction should be performed. What proportion you choose for duplicate extraction or checking depends on time/resource constraints, but this step is very important for quality assurance.

## Discussion

Although there are previous examples of guides and forms available for evidence synthesis [2, 3], these are aimed more at those wishing to perform data extractions for standard pairwise meta-analyses. Currently, no such guide exists for more complex evidence synthesis techniques, such as NMA or multivariate meta-analyses, which often require larger and more complex data extractions.

The DECiMAL guide aims to address this by providing a series of relevant suggestions for how to improve data extraction for complex meta-analysis, supporting the suggestions for how to extract different types of data with several different examples. It is intended to help support reviewers when embarking on a complex meta-analysis and to prepare them in advance for situations they might encounter during data extraction that might lead to inconsistency in the way results are extracted and coded. It does not provide advice on good statistical practice but suggests steps to ensure that sufficient information is extracted to allow any type of analysis (e.g. missing data using either complete case analysis or imputation).

Results from the pilot study showed that the guide was both easy to learn and useful, though the type and format of data to be extracted can add complications when developing a data extraction template. Reviewers found that whilst the DECiMAL guide gave them useful advice in a form that was easy to refer to whilst working, starting a complex data extraction without support from someone with experience was challenging, and the guide could not be a replacement for technical expertise.

## Conclusions

We propose that the guide should be read by reviewers before designing data extraction forms and embarking on the data collection process and should be kept handy throughout the process, in case some studies report data in a format the reviewer is not so familiar with. We expect that this will be most useful for reviewers who may be experienced in extracting data of a certain type (e.g. continuous data for pairwise meta-analysis), but who are now faced with extracting different data, for a different type of analysis (e.g. rate data for network meta-analysis).

The generalizability of these instructions across different data collection programmes and the potential benefits of a well-conducted data collection make this guide a valuable resource for anyone about to embark on any type of statistical analysis resulting from a systematic review.

## Additional file

Additional file 1: Excel workbook containing example data extractions for different analyses and types of data as described in the DECiMAL guide. The workbook contains the following worksheets - One study per row (arm), One study per row (relative), Rate data, Diagnostic test accuracy, Codebook. (XLSX 26 kb)

## Abbreviations

NICE: National Institute for Health and Care Excellence
NMA: Network meta-analysis
NR: Not reported
SD: Standard deviation
SE: Standard error
